# Semantic modelling of common data elements for rare disease registries, and a prototype workflow for their deployment over registry data

**DOI:** 10.1186/s13326-022-00264-6

**Published:** 2022-03-15

**Authors:** Rajaram Kaliyaperumal, Mark D. Wilkinson, Pablo Alarcón Moreno, Nirupama Benis, Ronald Cornet, Bruna dos Santos Vieira, Michel Dumontier, César Henrique Bernabé, Annika Jacobsen, Clémence M. A. Le Cornec, Mario Prieto Godoy, Núria Queralt-Rosinach, Leo J. Schultze Kool, Morris A. Swertz, Philip van Damme, K. Joeri van der Velde, Nawel Lalout, Shuxin Zhang, Marco Roos

**Affiliations:** 1 Leiden University Medical Center, Leiden, The Netherlands; 2 Departamento de Biotecnología-Biología Vegetal, Escuela Técnica Superior de Ingeniería Agronómica, Alimentaria y de Biosistemas, Centro de Biotecnología y Genómica de Plantas (CBGP), Universidad Politécnica de Madrid (UPM), Instituto Nacional de Investigación y Tecnología Agraria y Alimentaria (INIA), Pozuelo de Alarcón, Madrid, ES Spain; 3 Department of Medical Informatics, Amsterdam Public Health Research Institute, Amsterdam UMC, University of Amsterdam, Meibergdreef 9, Amsterdam, The Netherlands; 4 Department of Medical Imaging, Radboud University Medical Center, Nijmegen, The Netherlands; 5 Centre for Molecular and Biomolecular Informatics, Radboud University Medical Center, Nijmegen, The Netherlands; 6 Institute of Data Science, Paul-Henri Spaaklaan 1, Maastricht University, 6229EN Maastricht, The Netherlands; 7 Division of Paediatric Nephrology, Centre for Paediatrics and Adolescent Medicine, University of Heidelberg, Heidelberg, Germany; 8 University of Groningen and University Medical Center Groningen, Genomics Coordination Center and Department of Genetics, Antonius Deusinglaan 1, 9713 AV Groningen, The Netherlands; 9 Duchenne Parent Project, Veenendaal, The Netherlands

**Keywords:** FAIR data, Rare disease, Interoperability, Linked data, Data transformation, Semantic web, Ontologies, Common data elements, Disease registries

## Abstract

**Background:**

The European Platform on Rare Disease Registration (EU RD Platform) aims to address the fragmentation of European rare disease (RD) patient data, scattered among hundreds of independent and non-coordinating registries, by establishing standards for integration and interoperability. The first practical output of this effort was a set of 16 Common Data Elements (CDEs) that should be implemented by all RD registries. Interoperability, however, requires decisions beyond data elements - including data models, formats, and semantics. Within the European Joint Programme on Rare Diseases (EJP RD), we aim to further the goals of the EU RD Platform by generating reusable RD semantic model templates that follow the FAIR Data Principles.

**Results:**

Through a team-based iterative approach, we created semantically grounded models to represent each of the CDEs, using the SemanticScience Integrated Ontology as the core framework for representing the entities and their relationships. Within that framework, we mapped the concepts represented in the CDEs, and their possible values, into domain ontologies such as the Orphanet Rare Disease Ontology, Human Phenotype Ontology and National Cancer Institute Thesaurus. Finally, we created an exemplar, reusable ETL pipeline that we will be deploying over these non-coordinating data repositories to assist them in creating model-compliant FAIR data without requiring site-specific coding nor expertise in Linked Data or FAIR.

**Conclusions:**

Within the EJP RD project, we determined that creating reusable, expert-designed templates reduced or eliminated the requirement for our participating biomedical domain experts and rare disease data hosts to understand OWL semantics. This enabled them to publish highly expressive FAIR data using tools and approaches that were already familiar to them.

## Background

The FAIR Principles [1] aim to provide guidance that will lead to an internet of data and services that is highly descriptive and machine-accessible, resulting in more extensive data discovery and reuse. FAIR (Findable, Accessible, Interoperable, and Reusable) data requires unambiguously identified entities to be richly described by unambiguously defined and identified concepts from thesauri and ontologies that are widely shared within a community and machine readable. When this is achieved, it will become much more straightforward to discover task-relevant data over distributed sites, accurately integrate those data, or analyse them by ‘data visiting’.

A significant barrier to Rare Disease (RD) research is that RD data is (a) extremely scarce, and (b) spread over many “boutique” repositories, often single-disease-specific and often curated by biomedically-oriented experts, who may not have access to experts in data or knowledge representation, capture or archival. In an initial step to address this, the EU RD Platform has begun to establish standards for integration and interoperability. The first practical output of this effort was a set of 16 Common Data Elements that should be implemented by all RD registries [2]. These include facets such as “sex”, “date of birth”, “age of onset”, and “diagnosis”, often together with a constraint on the allowed values of each of these data elements (for example, the possible values of ‘age at onset’ are ‘Antenatal’, ‘At birth’, ‘Date (dd/mm/yyyy)’, or ‘Undetermined’). Achieving uniformity of these 16 data facets, over all RD registries and biobanks, would be an excellent first-step towards enhanced discovery and reuse of these precious data. Web-scale – which implies “mechanized” – interoperability, however, requires decisions beyond just a list of data elements, including data models, formats, and semantics.

The European Joint Programme on Rare Diseases (EJP RD) is an expansive European (with foreign partners) project aiming to reduce the suffering of rare disease patients and their families, through technical, clinical, social, economic, and health-services mechanisms. EJP RD spans 35 countries with 87 beneficiaries and 52 linked parties, and spans all 24 European Rare Disease Reference Networks (ERNs), totalling approximately 1200 people. Each ERN focuses on a particular class of rare diseases (for example, neuromuscular or vascular), and thus each ERN will have multiple registries and/or biobanks, which will vary in their level of systematic and schematic coordination even within a single ERN. To improve the utility of this massive ecosystem of data collection and curation, EJP RD aims to further the goals of the EU RD Platform by generating a “Virtual Platform” for interoperability between RD data assets throughout Europe and beyond. Speaking only of the technology layer, the Virtual Platform will provide common, harmonized access to discovery of task-relevant data resources, supported by a rich layer of metadata describing the content and context of each participating ERN repository. In part, this is being pursued by generating metadata that follows the FAIR Data Principles [3, 4], and global metadata standards are well-established (e.g., Dublin Core [5] and Data Catalog – DCAT [6]). This cannot be said for data, however. The diversity of data, and the wide range of mechanisms, tools, and devices for generating it often thwart generic approaches to creation of data schema. Nevertheless, historically, within the RD community, there have been efforts to train individual data custodians to create FAIR data at-source. These have taken the form of annually recurring “Bring Your Own Data” [7] workshops (BYODs) where data custodians meet FAIR experts and get hands-on experience in making their resources FAIR.

Because of their open-ended, exploratory structure, these BYOD events did not converge on a unified model for RD data, nor even the elements that should be included in those models. As such, the workshops primarily succeeded in raising awareness of FAIR, and the utility and benefits of following the FAIR Principles; however, the degree of inter-repository harmonization achieved by these workshops was extremely limited. Nevertheless, some preliminary data models [8] were created at BYOD workshops, including the early version (V0.1.0) of CDE semantic model that is the focus of this manuscript, which was developed during the FAIRification of a registry for vascular anomalies [9–11]..

In the case of the EJP RD project, it was immediately clear that training individual participants in FAIR data modelling themselves would be challenging for many reasons - RD registries are limited by funding, FAIR expertise, and time. All three of those barriers make it infeasible for the initial FAIRification pathway for EJP RD to involve significant decision-making by the resource custodian. Rather, we decided to centralize many of the decisions, ensuring that they were made by a small group of FAIR experts, and then disseminated outward to the individual participating registries and biobanks via a layer of registry “liaisons” who would communicate the needs, in both directions, between the data modellers and the registry custodians.

The final problem was how to enact the FAIRification itself - that is, how to do the “extract” and “transform” portions of the traditional Extract/Transform/Load (ETL) pipeline over resources that had no coordinating structure, and potentially no ability to code data transformation software themselves. Thus, we needed to identify an ETL pipeline that could be deployed anywhere, over any native data structure, in highly secure privacy-sensitive environments, and execute a successful transformation using only the expertise that could be expected of most repository curators.

Here we describe the process of data modelling within the EJP RD, as applied to the set of CDEs defined by the EU RD Platform. We describe the semantic basis of those models, and how they have already been applied to distinctly different data types, showing that they have not been overly “fitted” to the data elements defined by the CDEs. Finally, we describe our current attempts to build an ETL pipeline that can fill these models, using a simple, structured Comma-Separated Value (CSV) export of source data from the originating registry hosts. To help orient a broad range of readers, we attempt to split the discussion into three groupings: “FAIR Expert Activities”, where technical details and decisions are described; “Data Custodian Activities”, where less technical deployment decisions and activities are discussed; and where relevant, “Data Steward Activities” where the role of the “liaisons” between the FAIR experts and the data custodians are highlighted.

## Methods

### FAIR expert activities - modelling

Modelling activities were undertaken via weekly meetings of a core group of EJP RD researchers with extensive experience in ontologies, knowledge representation, Linked Data modelling, and FAIR data. Meetings were carried out via Microsoft Teams, where the model under discussion was presented via screen sharing.

As noted above, the European Platform on Rare Disease Registration has determined a set of 16 CDEs for RD registration. These are detailed in Table 1.

**Table 1:** The European Platform for Rare Disease Registration set of Common Data Elements that should be made available by all rare disease registries

Element ID	Name	Values
1.1	Pseudonym	String
2.1	Date of birth	dd/mm/yyyy
2.2	Sex	Female, Male, Undetermined, Foetus (Unknown)
3.1	Patient Status	Alive, Dead, Lost in Follow-up, Opted-out
3.2	Date of Death	dd/mm/yyyy
4.1	First contact with specialized centre	dd/mm/yyyy
5.1	Age at onset	Antenatal, At Birth, Date (dd/mm/yyyy), Undetermined.
5.2	Age at diagnosis	Antenatal, At Birth, Date, Undetermined
6.1	Diagnosis of the rare disease	ORPHA Code, Alpha Code, ICD9/10 Code, ICD9-CM Code
6.2	Genetic Diagnosis	Human Genome Variant Sequence (HGVS), HUGO Gene Nomenclature Committee (HGNC), Online Medelian Inheritance in Man (OMIIM) Codes
6.3	Undiagnosed case	Human Phenotype Ontology code and/or HGVS Code related to the inability to diagnose.
7.1	Agreement to be contacted for research purposes	Yes/No
7.2	Consent to reuse data	Yes/No
7.3	Biological Sample?	Yes/No
7.4	Biobank?	URL/No
8.1	Disability Classification via International Classification of Functioning and Disability (ICF)	Score

Using these elements as a guide, together with additional documentation detailing how these elements should be filled, a first pass modelling phase [9] was undertaken where Linked Data representations for each CDE were constructed, using existing ontological terms or other shared Globally Unique Identifiers (GUID) wherever possible to model, for example, genotypes (OMIM Codes [12]) and phenotypes (Human Phenotype Ontology Codes [13]).

These first-pass models were then used to frame a more conceptual modelling process, looking at (for example) the inter-dependencies between the CDEs, the “nature” of the data – for example, is it obtained by questionnaire or by physical examination? – and what additional annotation would be useful to contextualize the CDE for correct interpretation (e.g., the dates of various phenotype onsets could be used to build a longitudinal record of the patient’s response to treatment). There were several over-arching guidelines that constrained this modelling process:

1) We should use the minimum number of ontologies possible.

2) We must strictly adhere to the ontological definition of a concept.

3) The ontologies/vocabularies used must not have a restrictive license.

4) The model should be designed in a forward-looking manner, anticipating other likely data elements, to minimize the need for future disruptive changes.

Examination of the EU RD CDEs revealed that there were, in fact, many inter-dependencies between them – meaning that one CDE could not reliably be understood or contextualized without one or more of the others. For example, CDE 1.1 - the patient pseudonym – must be a part of every CDE, since all CDEs are related to an individual patient. For example, 4.1 ‘First contact with a specialized centre’ cannot be interpreted without a reference to the patient that made contact with the centre (via their pseudonym). Similarly, since individuals may have multiple diseases, each with its own diagnosis (CDE 6.1), the “age at diagnosis” (CDE 5.2) must somehow relate to the disease which was diagnosed at that age. In addition, we noted that most CDEs focused on data that would result from a formal interaction in a clinical setting, but those data gathering processes might be undertaken at different locations and times. For example, obtaining a biological specimen (CDE 7.3) would often be a surgical process, which would be undertaken in entirely different circumstances than the administration of a questionnaire to generate a disability score (CDE 8.1). Although the CDE requirements from the EU RD Platform do not require that this metadata be represented, it is nevertheless true that these details likely are being captured in many cases, thus it is useful to plan a model that can carry this contextual metadata, now or in the future. This minimizes the degree to which EJP RD participants would have to change their workflows to adapt to future changes.

We examined two ontologies that are commonly used in the life sciences to model processes, workflows, and their participants. The Provenance ontology (PROV-O) [14] aims to capture information about the sequence of events that leads to an output, such as who executed which version of which algorithm at what time, using what input data, and where is the output data stored. The Semantic Science Integrated Ontology (SIO) [15] is also capable of modelling entities, processes, and their qualities/attributes, but includes additional entity-to-entity or entity-to-process relationships that enable rigorous and highly explicit machine-readable patterns associating these to one another. SIO also has domain extensions, including biology and bioinformatics, that can help ensure that many clinical or biological concepts are being used in a logically sound manner. For example, SIO includes CDE-relevant concepts such as “medical diagnosis”, which allows us to use SIO-defined properties and entities for the majority of the CDEs. Finally, SIO has the capacity to represent data content – that is, while PROV-O has the concept of an Entity, which could represent the output of a process, it does not have the ability to describe what that entity is, or its value, or its measurement units. Finally, PROV-O has no way of representing the attributes or qualities of an individual. Given that almost all of the CDEs are measurements of some attribute of the patient who participated in a clinical process, the inability to associate the output of a process (like a phenotype) as an attribute of the participating patient would make a PROV-O model highly unintuitive for our target end-users. As such, while PROV-O might be useful at a later date to describe, for example, the precise details of a Phenotyping or Genetic Diagnosis workflow, our needs in this modelling exercise are distinct, and are better represented by SIO concepts.

**Fig. 1:**
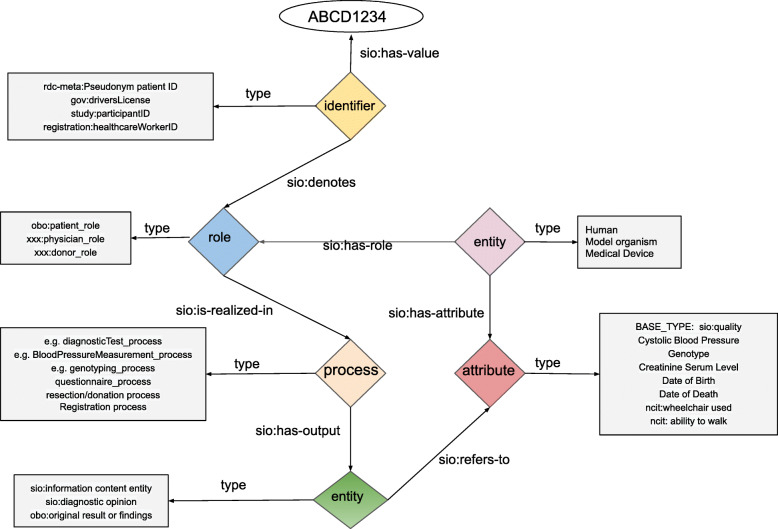
Conceptual diagram of the overall SIO model to be applied to the CDEs. It is centred around five primary elements – identifiers, entities (physical and information-content), roles, processes, and attributes. In the diagram, we provide hypothetical examples of the specific ontological types that might be associated with each element

Following the documented design patterns for SIO [16] we derived the core model shown in Fig. 1. Some of the rationale for this model are as follows: All CDE observations are, in some way “about” an individual patient. As such, it is necessary to connect patients to these observations. In some cases, the CDEs pertain to a direct attribute of the patient (e.g., their birth date). In other cases, the CDE is not an attribute of the patient per se, but rather the connection between a patient and the CDE is via an action or activity that the patient engaged in; for example, the first interaction of a patient with a rare disease expert centre. Certainly, for all CDEs there is at least the process of recording the information, and as such, we decided that a “process” was a concept shared by all CDEs. Early discussions also raised the issue of an individual having multiple roles in the healthcare system, for example, being both a patient and a physician. As such, it was necessary to connect a participant to the process indirectly, by declaring the role they play in the process. An individual may have many roles, and we determined that in every case, there was a distinct identifier that was assigned to that role – for example, a driver’s license ID is assigned to one’s role as a driver, and a student ID is assigned to one’s role as a student, yet both identifiers may apply to the same individual. As such, we associated identifiers with individuals via their role, rather than directly. Finally, processes have outputs, where those outputs (often) refer to some measurement of an attribute of the patient. The attribute, and its measurement, are distinct – for example, all patients share the attribute of “sex” but for some patients this attribute has the value “male” and for others it has the value “female”.

Combining these considerations leads to the core model shown in Fig. 1, where there are 5 “kinds” of things: entities (individuals, and measurements), roles, processes, attributes, and identifiers. While there are additional relationships between these concepts, we removed all but the relations required to connect the model. This will simplify the creation of query systems, by limiting the possible ways the model can be explored, better enabling the construction of reusable query templates (an activity that is also being undertaken within the Virtual Platform, to ensure that end-users do not need to learn a formal query language for these data).

Using this high-level model as a guide, the EJP RD semantic modelling group then reiterated the process of examining each CDE and, through Teams meetings and dedicated “designathons” we reached agreement on which portions of the high-level model were appropriate for each CDE, and what the ontological type constraints (square boxes in Fig. 1) should be for the elements of that specific CDE model. As part of the modelling process, we selected a “base type” for each of the model elements, for example, the process node is always ontologically typed as a “sio:process”. In this way, if there is not a more specific type assigned to the model node, we still maintain the best practice of having all nodes in our model ontologically typed. These “base types” are built into our transformation templates (described below) and require no knowledge by the end-user. Finally, where appropriate, we selected ontological concepts that would be allowed as values or attributes for various CDEs. For example, in the personal information CDE we selected the National Cancer Institute Thesaurus’ [17] terms for “male” and “female”, and we selected the Human Phenotype Ontology [13] as the set of possible values for the phenotypic diagnosis CDE.

### FAIR expert activities – design a “lingua franca” for data extraction

Registries participating in the EJP RD have a wide range of underlying infrastructures and data management and curation expertise, ranging from well-established commercial enterprises such as Castor [18], to smaller, parent-run organizations even using spreadsheets to capture data. They are also largely not coordinating with one another and are therefore making independent decisions about database structures and the formats of the captured data. It was therefore clear that, as an initial step to harmonization, we needed to find a “lowest common denominator” for an intermediate data representation format – something more predictable than the various source data structures, but not yet FAIR. It needed to be a format that can be generated by data custodians at any level of expertise, from any starting format, hopefully using only tools with which they are already familiar. Moreover, a primary objective of EJP RD is to encourage FAIRness beyond the EJP RD itself, thus the selected format should be applicable to a wide variety of expert domains and situations.

Through discussions with EJP RD partners, it became clear that there was a preference for very straightforward data structures such as CSV, since this is an exchange format that can be derived easily from any of the more complex formats. As such, CSV was selected as the “lingua franca” that would be used by all participants as an export format, as a step towards harmonization and FAIRness. The rules given to the data custodians explaining how to generate these CSV files is described in the section “**Data Custodian Activities – Generating template-compliant CSV**”.

### FAIR expert activities – CSV to RDF mapping

Having selected CSV as a starting format, we then chose a mapping framework that could transform CSV data into RDF. RDF Mapping Language (RML) was the selected technology, as it is capable of modelling reusable templates that support not only CSV to RDF transformations, but also transformations from other formats, allowing us the opportunity to increase in complexity in the future without dramatically changing our pipeline.

RML templates specify individual triple patterns that should be created during a transformation. The subject Uniform Resource Identifier (URI), predicate URI, and object URI are represented as strings that may contain variables, where the variables are references to locations within the source document (e.g., the appropriate column header within a CSV file). During a transformation, every variable in an RML template is replaced by the value of that location within a single source record (e.g., a single row of a CSV file) and then the source is iterated over all records to complete the transformation. RML templates themselves are represented in RDF and are therefore not always easily human-readable. With the aim of simplifying the RML syntax, such that our EJP RD FAIRification stewards, or potentially the registry data custodians themselves, could edit the template if required, we identified a second, related technology – YARRRML [19] – which is a more human-readable way to declare RML transformation rules, using YAML as the syntax. YARRRML documents can be converted into RML templates, which can then be automatically applied to CSV files to achieve their transformation. Having selected these tools, the FAIRification experts then transcribed each of the CDE models into a set of YARRRML rules that were then executed to generate RML mapping documents. The YARRRML documents are stored in the “YARRRML_Transform_Templates” folder of the CDE Project GitHub [20] for others to explore and reuse. The final step of modelling was to create documentation and example data to provide to the registry custodians, to guide them in the requirements for the CSV files.

### Data custodian activities – generating template-compliant CSV

The process for creation of the CSV files for each CDE will likely differ for each registry, as their individual situations will be diverse. Based on the documentation and examples provided by the FAIR Expert team, considerations for the registry custodians include:

- Ensuring date formatting is correct (ISO 8601)
- Ensuring that any abbreviated ontology terms have been converted into their equivalent full URIs (e.g., Human Phenotype Ontology terms must be represented by their URI in the CSV file)
- Ensuring any data elements have been modified to match the documented constraints (e.g., conversion of textual descriptors into ontology term URIs)
- Ensuring that every row is uniquely identified (for this purpose, we have established a web service that can be called from MS Excel or a custom script that generates a unique identifier based on a timestamp, since MS Excel has no inherent capability to generate GUIDs without custom coding)

### Data steward activities – assisting data custodians

In anticipation of the data custodians having questions about how to generate template-compliant CSV and the rationale behind certain decisions, every participant was assigned a FAIR Data Steward to provide them with assistance and advice. For example, we could anticipate data custodians being concerned about why one ontology was chosen over another or needing advice on a tool that can cleanse or edit CSV files. In addition, the FAIR Stewards would bring back suggestions from the Custodians to the FAIR Expert meetings, providing a useful feedback mechanism where the Steward had personally engaged with the data, and understood the concern, as well as being a FAIR expert themselves who could relay the concern accurately to the FAIR Expert team.

### FAIR expert activities – building a transformation pipeline

“RDFizing” is the process of transforming a non-RDF data format into RDF. We tried two RDFizers that execute such transformations using RML as the mapping language – RMLMapper [21], and SDM-RDFizer [22]. RMLMapper has a rich set of features, including the ability to encode transformation rules that can trigger execution of algorithms over a CSV cell prior to the RDF transformation. SDM-RDFizer conversely, lacks these powerful extensions, but is significantly faster in our (informal) head-to-head tests. Since YARRRML currently cannot encode rules, we do not benefit from the additional power provided by RMLMapper, and thus selected SDM-RDFizer for this modelling initiative. Nevertheless, the choice of RDFizing technology can be revisited at a later date, without affecting any of our other decisions.

For storage of the resulting Linked Data, we have selected GraphDB [23], due to its ongoing support by the developers, the availability of a free (though not open source) version, and the availability of a fairly comprehensive API for mechanization of data loading, maintenance, and querying. GraphDB also supports access control methods which provide options for securing access to the FAIRified dataset. A “bootstrapping” Docker image for GraphDB was created to ensure that GraphDB is installed and configured correctly, thus eliminating the need for the registry host to have this expertise.

Deployment of the ETL pipeline is achieved via docker-compose, where every component has been “dockerized” and uses a Docker [24] network to facilitate communication between the components. This ensures that there are no unnecessary ports or APIs exposed on the registry server, helping maintain the security of their internal space. The three components mentioned above - RMLMapper, SDM-RDFizer, and GraphDB - are coordinated via a fourth Docker container, representing an orchestration tool. The orchestrator is triggered by a Web call to its interface. Once initiated, it automatically refreshes the current database of YARRRML templates from the CDE Project GitHub, and then examines the content of a folder shared with the host. This shared folder contains the host’s CSV files that will be subject to RDF transformation. Using filename-matching, the system matches each CSV with an appropriate YARRRML template and executes the transformation. After all transformations have completed, a connection is opened to GraphDB, all previous data is deleted, and the refreshed data is uploaded.

The suite of docker images are referred-to as the “CDE-in-a-Box”, and the instructions for running the bootstrapping process, as well as how to interact with CDE-in-a-Box, are available on a dedicated Git [25].

### FAIR expert activities – testing

Speed tests were run by calling RMLMapper and SDM-RDFizer images via docker-compose on a Linux PC. A variety of exemplar 10.000 row CSV files and YARRRML templates were used for the measurement and execution process. The average speed of RDF triple generation was 12,500 triples per second. The tests were run on an AMD Ryzen 73800XT 3.9 GHz CPU workstation, with 32 Gb 3200Mhz RAM memory, RTX 2070 Super 8 Gb GPU and M.2. NVMe SSD memory. Quality-control tests will, largely, be registry-specific, though we are considering possible mechanisms for generalizing this problem through the use of Shape Expressions (ShEx) validation (described in “**Future Work**” in the Discussion section).

## Results

### The models

The models created to capture the 16 CDEs are described in Table 2, and are available in the CDE Project GitHub .

**Table 2:** Models created to represent the CDEs. Models are created in YARRRML and made available on the CDE Project GitHub, accompanied by markdown documentation explaining the structure of an appropriate CSV file. Note that not all EU RD CDEs appear 1-to-1 with a CDE model. This is because, for example, the consent CDE can be reused for diverse types of consent (e.g., consent for contact, consent for data reuse), and the Pseudonym CDE is a part of every other model, and therefore has not been modelled as an independent element

CDE Model Name	Purpose
Disease Progression [26]	A “container” node to group together all other CDEs that refer to the same diagnosis. For example, the “age of diagnosis” CDE is related to a specific rare disease via traversal into the “disease progression” container, and then traversal into the “diagnosis” CDE that is also connected to “disease progression”
Care Pathway [27]	Captures the date of first contact with the specialist healthcare system; is connected to “disease progression”
Diagnosis [28]	Captures the final disease diagnosis using ORPHA codes; is connected to “disease progression”
Disease History [29]	Captures age at first symptoms and age at diagnosis; is connected to “disease progression”
Genetic Diagnosis [30]	Captures the sequence variant(s) found in this patient, using a variety of different coding systems; is connected to “disease progression”
Patient Consent [31]	Captures the consent of the patient over several axes (e.g., consent for contact, consent for data reuse). Provides a reference to the signed consent form, as well as an input reference to the (blank) consent template.
Patient Status [32]	Captures the current status of the patient, and their date of death if the patient is deceased
Personal Information [33]	Captures (superficial) personal information such as birth date and sex (there are ongoing debates in the EJP modelling group as to whether this should be converted to an age, or an age-range, for improved privacy)
Phenotyping [34]	Captures the phenotypes of the patient, using Human Phenotype Ontology terms
Disability [35]	Captures the score for a disability test. The specific test administered is indicated as one of the child nodes of obo: NCIT_C20993 (Clinical or Research Assessment Tool), and thus this CDE model is broadly useful for many disorders.
Undiagnosed [36]	Captures the case where a patient has phenotypic anomalies, and an identified sequence variant, but for some reason has not been definitively diagnosed.

**Fig. 2:**
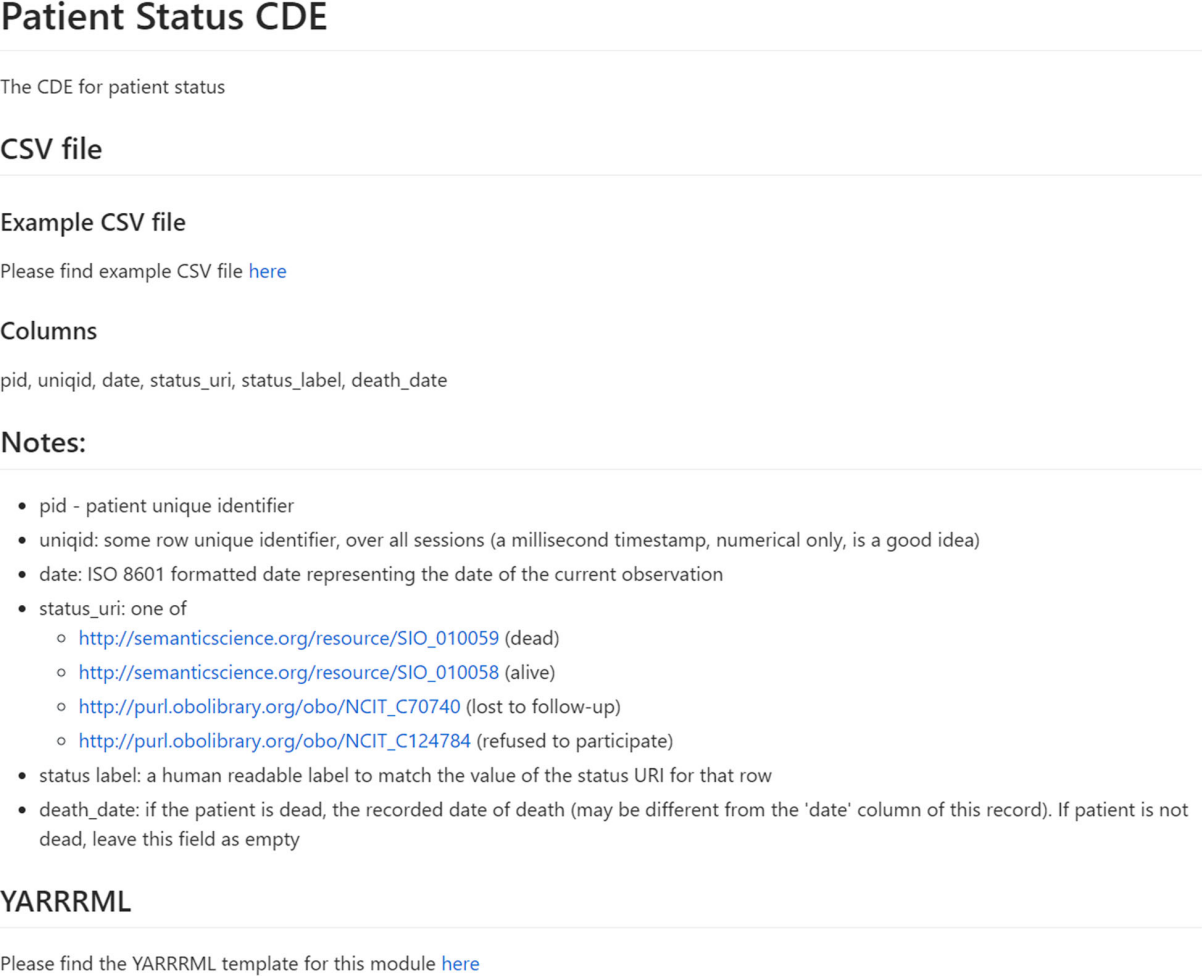
The Markdown documentation explaining how to prepare a CSV file for the “Patient Status” CDE. Documentation includes, where appropriate, the restrictions on the possible values in a given column, such as ‘status uri’ in this example

To help data custodians understand the models, they are generated and published in a variety of formats. Most importantly, an exemplar “runnable” CSV file, which is documented on a Web page – one page per CDE – containing a description of the CDE Model, its intended use, the CSV column headers, the constraints on the content of each column, and any usage notes that will assist the data custodians in their understanding of how to generate compatible CSV. A screenshot of the documentation is provided in Fig. 2.

**Fig. 3:**
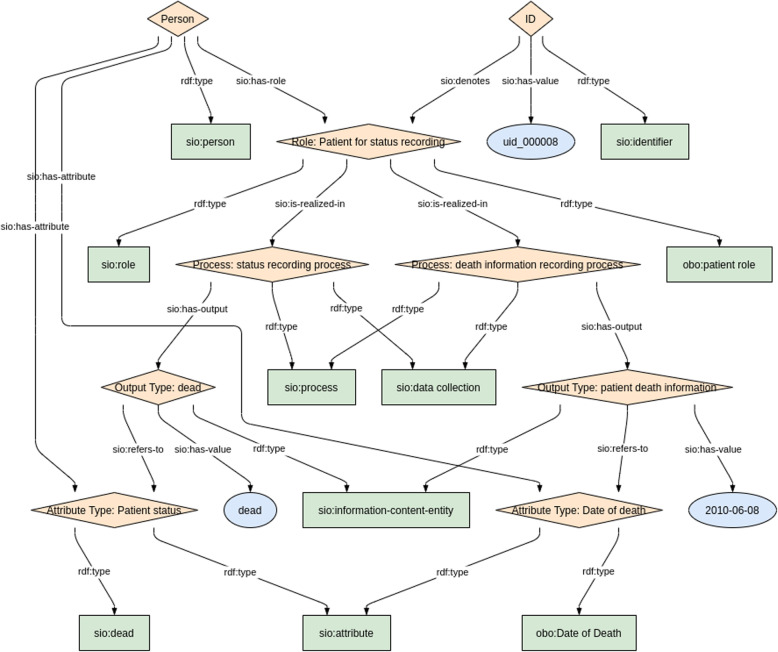
Visualization of an exemplar RDF instance for the “Patient Status” CDE (CDE 3.1 & 3.2)

**Fig. 4:**
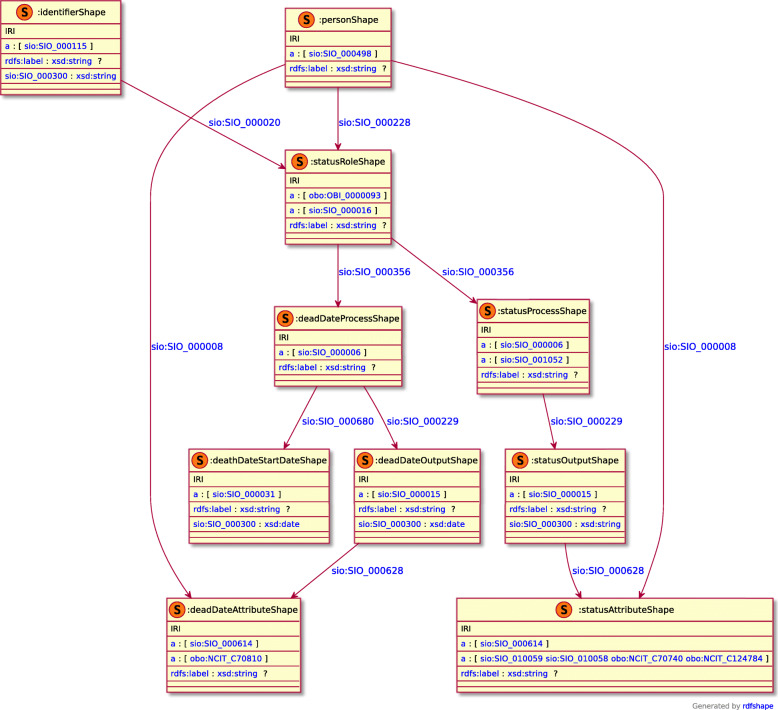
Visualization of the ShEx validation shape for the Patient Status CDE data

To assist both data custodians and data consumers, a variety of other representations are also generated. When the exemplar CSV is run through the transformation pipeline, the resulting RDF file is then converted into a model image via a semi-automated mechanism [37]. A ShEx model is also created to allow data custodians (and users) to validate these transformations. The ShEx models are manually created according to the Shape Expressions 2.1 Primer specification [38], and the resulting ShEx file is converted into an image via the RDFShape tool [39]. An exemplar RDF visualization for CDE #3 “Patient Status” is diagrammed in Fig. 3, and a diagram of the ShEx validator for that model is shown in Fig. 4.

### Model filling - “CDE in a Box”

As described in the Methods section, the CDE-in-a-Box is deployed via docker-compose and is triggered by a simple Web call to a local address. Transformed data is automatically loaded into a CDE-specific data store on GraphDB, ensuring that the security constraints on this data can be managed independently of other datasets provided by the software.

**Fig. 5:**
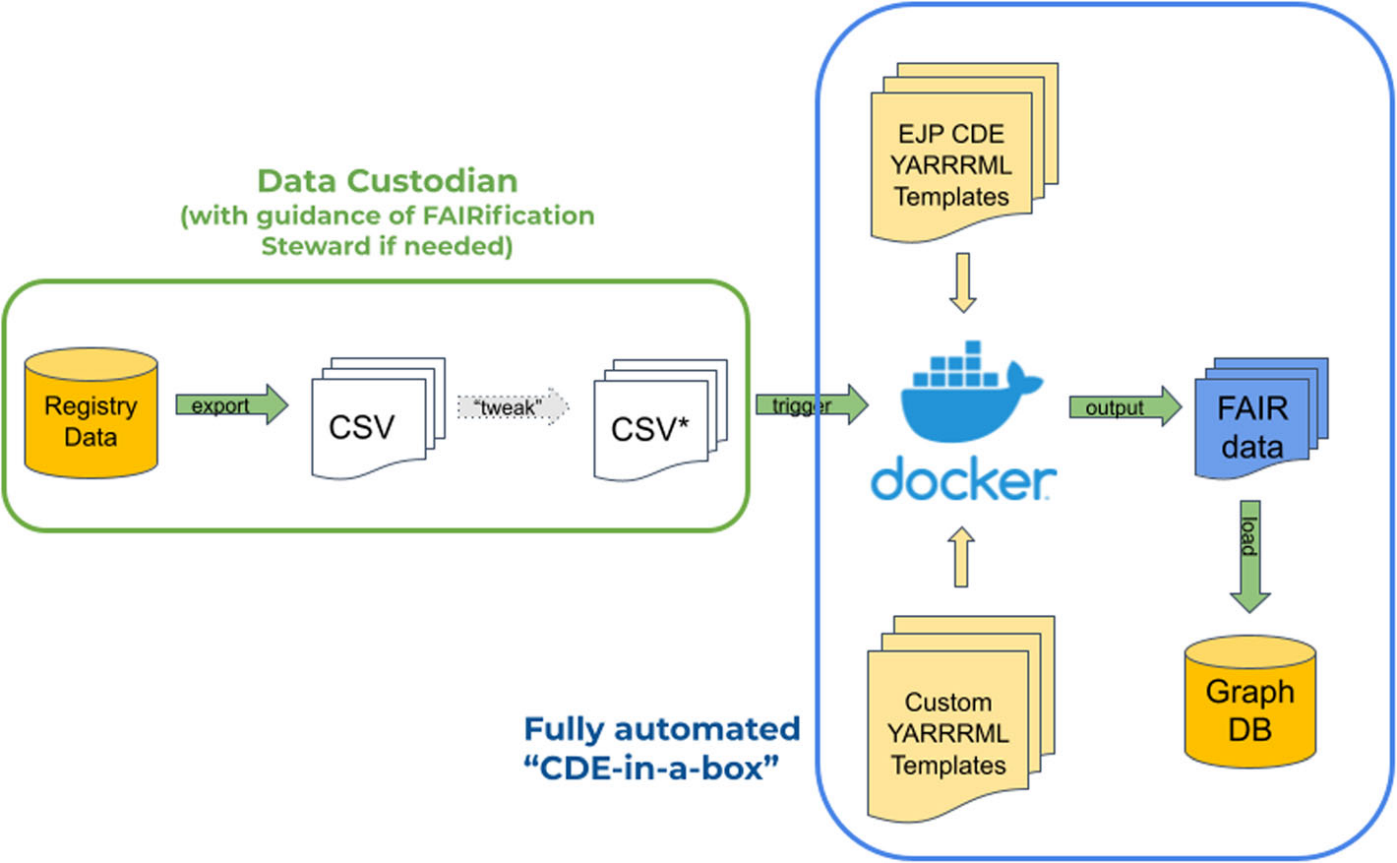
The components of the workflow annotated with the responsibilities of the parties. The left side of the diagram, outlined in green, are the responsibilities of the data custodian in collaboration with the Data Steward. This includes export of their registry data into CSV format, and possibly some additional modification of that exported data to conform to the template. On the right is the fully automated CDE-in-a-Box, which is constructed by the FAIR Expert team and provided as a docker-compose installation. The arrow labelled “trigger” is the Web page call that the data custodian makes when they are ready to execute their transformation

The relationship of all of the components to one another, and the responsibilities of each participant, is diagrammed in Fig. 5.

## Discussion

**Fig. 6:**
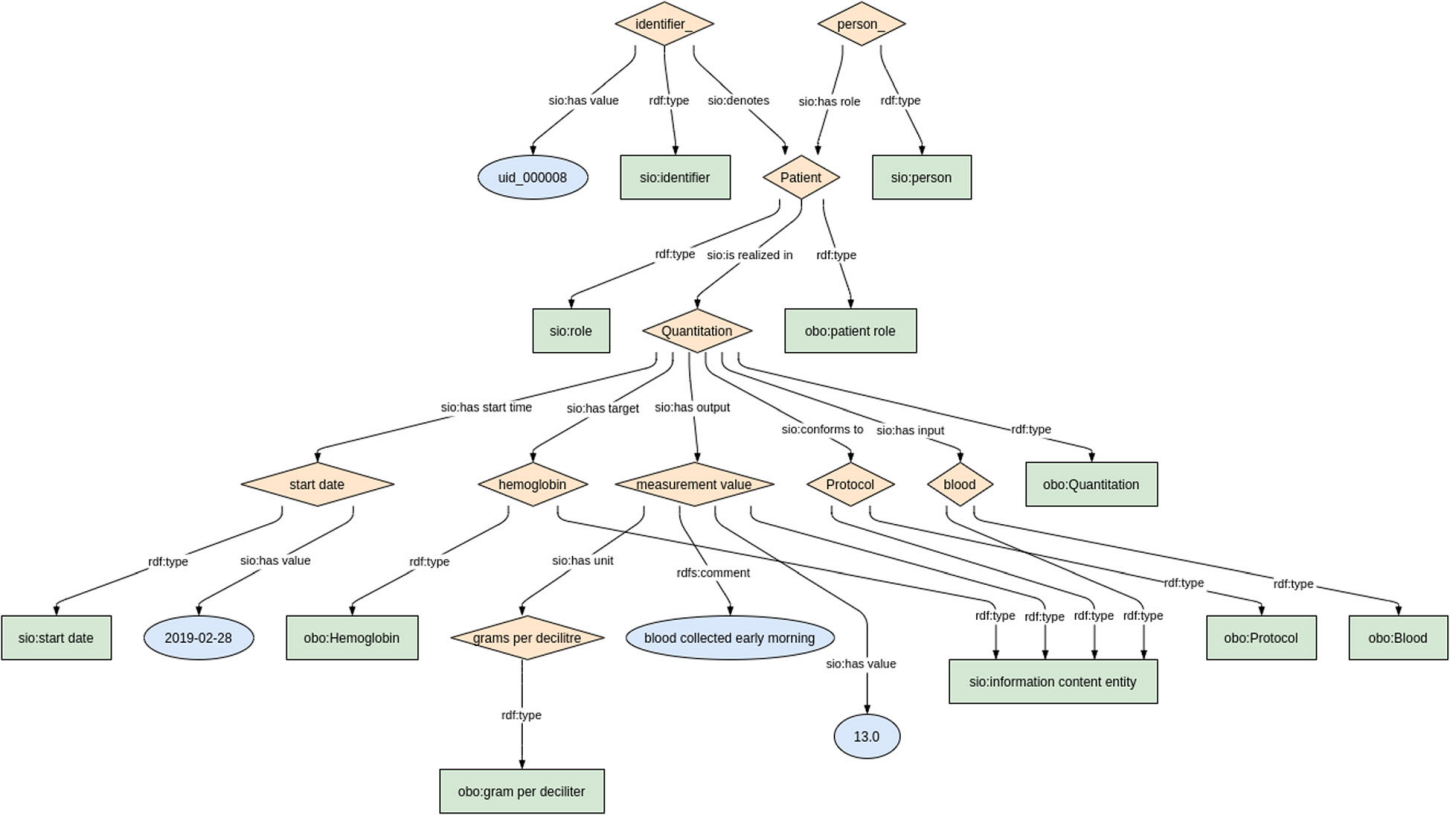
The model for Laboratory Measurements. Of note are the three new connections on the “Quantitation” (Process) node – one representing the input (blood), one representing the target molecule (haemoglobin), and the third representing the link to the protocol. The remainder of the model is (structurally) identical to the core model shown in Fig. 1

When undertaking any modelling activity, there is always the potential to “over-fit” the model. To this end, we have been attempting to apply the model to datatypes other than those covered in the CDE list. Specifically, we have looked at three very distinct datatypes: physical body measurements, laboratory tests, and Patient-Reported Outcome Measurements (PROMs), which are a questionnaire-style metric. In all cases, we were able to generate the Linked Data record with few or no changes to the core model. In particular, the Physical Body measurements required only an additional link to a measurement protocol; for PROMs we added an Input to the Process node representing the PROM question; and for Laboratory Tests we extended this further where an Input is included - constrained to being a body tissue - a “target” is included - constrained to being the compound being measured - and link is added to the measurement protocol document (see Fig. 6). Hence, we believe that this core model is capable of representing the majority of data entities we will encounter in the biomedical/clinical space with only minor modifications.

With respect to generalizability and scalability of this approach, a comprehensive survey of the European Reference Networks (ERNs) participating in EJP RD revealed 13 categories of data from 16 ERN data dictionaries; for example, “laboratory tests” and “personal information” are two such categories. Every category requires a YARRRML template to be constructed, following the core pattern but changing, for example, the default ontological types of each node, and the column header names. We have built code libraries that automatically generate these YARRRML templates via a simple API, and thus in practice, a new YARRRML model can be created in approximately one hour, now that the general pattern has been established.

Documentation of the model, and decisions about the constraints on the allowed content of each CSV column takes more consideration and time, though all elements of a well-documented model can be easily created in less than a day. This, however, leads to a problem for which the correct solution is, as yet, not known. Because the models themselves are generalized, the problem of selecting the correct specific value for a given column becomes a task for the data provider. For example, in the Body Measurement model, we document that the “attribute being measured” column should contain an ontology URI that is a child of obo: NCIT_C19332 (personal attribute). While many of the participating hosts are familiar with ontologies, “coding” (the act of assigning a controlled vocabulary term to a concept, observation, or phenomenon) is an activity primarily undertaken by insurance and governmental organizations, and by trained disease classifiers, and as such many other participants will not have this experience. Thus, we suspect that this task may be difficult for a subset of our registry participants. One alternative is that the FAIR Experts create a specific model for every case (every attribute, every lab measurement, etc.). This, however, would result in many highly specific models, and would in turn, require the data host to generate separate CSV files for each model. The alternative is to keep the models generic and find another way to provide advice or support to the data hosts as they generate the CSV. We are exploring both solutions to gain a better understanding of how to address this problem in the future.

The transformation step itself – generating ~ 12,500 RDF statements per second – would appear to be sufficiently fast that it would be possible to generate a new snapshot of a registry on a nightly basis.

Finally, the models are intended to be reusable, and the onus of creating a matching CSV is put on the data custodians/experts. Another approach would have been to create a comprehensive transformation map of the entire dataset, for every participating registry using, for example, R2RML [40]. Since the data custodians are not anticipated to be FAIR experts, this approach would have centralized the problem of mapping into the hands of the few core technologists in the EJP RD, which we feel is a much less practical solution, and less scalable. The solution proposed here distributes the effort over many more participants, and the sharing of a set of core models ensures that, despite being non-coordinating, the participating registries will nevertheless generate interoperable outputs.

### Exemplar use-case

Two registries about vascular rare diseases have entered into a data sharing agreement for a study on the relationship between identical mutations and phenotype/disability over many individuals. They select the Genetic Diagnosis, Phenotyping and Disability CDE as those that will contain the most relevant data. One registry executes three SQL queries on their Oracle database, which generate three CSV files following the CDE Model Templates. They activate CDE-in-a-Box, which converts those data into FAIR Data loads it into a database within their own secure space. The partner has their data in the form of a series of MS Excel spreadsheets. They export from spreadsheet into CSV, and similarly activate CDE-in-a-box to generate FAIR Data. The investigative query is shared by both sites, and executed within their secure spaces, where they exchange only the query results. They are confident that they are extracting and integrating the full gamut of information from both sites because of the harmonization of structural and ontological choices that are enforced by the CDE models, yet their individual tasks only involved generating the CSV and executing the query. Thus, the process of querying over both datasets was quite straightforward for both participants, despite having notably different underlying data structures.

### Peer initiatives

Beyond CDEs, the most widely used health care data exchange formats are all exploring FAIR-oriented mappings. OMOP CDM [41] and ContSys [42] were recently compared [43] for their ability to be transformed to one another, and to have their facet values captured in (largely) SNOMED CT to enhance their FAIRness. The HL7’s FHIR4FAIR project began its public facing activities at a “connectathon” event in early 2021 [44] and expects to have an early and final normative document in late 2023 and early 2024, respectively describing (among other things) how to apply the RDA FAIR Maturity Model Working Group Maturity Indicators [45] to FHIR data structures (both manual and automated) and define a minimal metadata set for health recordsets. The Critical Path Institute (C-PATH) [46] is working in parallel with the FAIR data transformation subgroup within the EJP RD to attempt to achieve a mapping between the Clinical Data Interchange Standards Consortium (CDISC) [47] standards and the EJP RD semantic CDEs, with their first attempt being to use LinkML/BioLink [48] as a domain-neutral abstract representation of the clinical data that might act as a “Rosetta stone”. Finally, the OpenEHR initiative has adopted design principles [49] that enables computable semantics in their data models [50]. Thus, the latter of these peer initiatives resembles our own attempts to build an overall model based in rich semantics; however, we differ from most of these peer initiatives in that we are attempting to map arbitrary existing formats from non-coordinating registries into a unified, semantically-grounded (in SIO) FAIR model, versus taking an existing standard model and attempting to make it more FAIR.

A notable peer initiative, with highly similar goals to our own, is found in PennTURBO [51], which also pursues a multi-step transformation process to achieve a final rich semantic model. PennTURBO’s workflow mirrors ours in several ways: first, there is a transformation from native format into a semantically-impoverished intermediate representation (in our case, CSV, and in PennTURBO, a graph). They then similarly use a domain specific language to transform the intermediate representation into the final semantically rich model (in our case, RML, and in PennTURBO, SPARQL). A key distinction between the two projects is the point of responsibility for the generation of the intermediate format. PennTURBO includes a set of data extraction/transformation instructions for each data source, thus the responsibility for the correct interpretation of the data source, and its export, is held by the PennTURBO development team. In contrast, the transformation pipeline presented here creates only a richly documented intermediate template, which must then be filled by any data source that wishes to participate. Thus, the responsibility for correctly filling that template is pushed to the participant themselves, as is the correct interpretation of the data holdings. It is an open question whether the decentralized approach we have taken will be more scalable than PennTURBO, or if the same decentralization will lead to more erroneous template filling, as the responsibility for accurate data export moves further away from the FAIR experts. Nevertheless, like PennTURBO, our mapping extends beyond identifying appropriate ontology terms for each data facet, and both projects share a goal of attempting to better model the activities around the creation of data – creating a “digital twin” for the data, which as a beneficial consequence, provides model positions for metadata about every element, including the participants, the relationships between them, and the process’ protocol and other annotations.

### Future work

Work is underway to automate the creation of ShEx models for all CDEs, and use them to add a quality-checking layer into the transformation pipeline. Moreover, we additionally plan to use ShEx to publish a public model of the entire contained dataset, which we believe can be used both to aid discovery, but more importantly, to facilitate future efforts around federated queries.

With the goal of allowing future extension of these models – for example, by expanding the ontological concepts allowed as possible values, or adding new or repository-specific metadata we will soon begin to provide training to those who wish to learn how to build or edit the YARRRML templates themselves. We are improving the tooling that facilitates construction of these templates to better enable registry custodians to expand or diversify the templates without necessarily requesting help from the EJP RD modelling team. In this way, we hope that the core data will be interoperable, even if individual sites add enhanced metadata that is not in-common with other registries. Moreover, dissemination of the expertise around template-building provides a path to self-sustainability of this initiative, beyond the end of the EJP-RD project.

Extension and revision, however, must be done with a recognition of why this centralized modelling initiative was deemed necessary. Interoperability is difficult to achieve, particularly without agreement on the concepts being modelled. Moreover, the decision to use a model backbone with very strict semantics (SIO) makes it necessary to be extremely careful in the selection of ontology terms – ensuring, for example, that there is a distinction between the concepts of “blood pressure” as a quality/attribute of a patient, versus “blood pressure” referring to the output of a measuring process. These kinds of decisions require expertise and experience in ontology construction and use. As such, extension of the models in a distributed manner by end-users introduces several risks regarding interoperability, including lacking mappings between ontologies, reduced shared semantics, and restricted use of ontologies, for example due to licensing restrictions. The same issue has been highlighted in other CDE initiatives, and was addressed in a recent overview of the problems related to CDE mapping [52] (using the term in its most general sense, not specifically the RD CDEs that are discussed in this manuscript). They noted that the objective of CDEs – to assist in the harmonization of data between independent studies – was being thwarted by imprecise definitions of those CDEs (a problem shared with the CDEs upon which this study is based). They further noted that “*CDEs can deliver more value when they conform to accepted data standards, are bound to terminologies and are used consistently across studies*”, and that, for this reason, CDE-focused initiatives are falling far short of the objectives of FAIRness.

## Conclusions

We undertook a process of constructing a reusable, generic data model, based on the design principles of the Semantic Science Integrated Ontology, to represent all the EU Rare Disease Platform Common Data Elements. Emergent mapping technologies such as YARRRML, RML, and “RDFizing” tools allowed us to create an automated pipeline for filling these data models starting from a well-documented CSV template – a format accessible to all our target end-users. We demonstrated the generic nature of the model by successfully extending it - while remaining within the overall architecture of SIO - to widely disparate non-CDE clinical data within the Rare Disease space. Feedback from end-users indicates that they found this solution helpful, and easy to apply. As FAIR data publishing becomes increasingly an expectation – even a requirement – of funding agencies and publishers, there is an urgent need for straightforward tooling to assist data providers to comply with these expectations. In many cases, those who generate data will not have expertise in data modelling, and particularly not in semantically grounded data modelling, as is a requirement of FAIR. The activities and workflows described here indicate that the approach of building a generic, reusable, models, and an automated pipeline to fill them, will be widely applicable in biomedicine and beyond.

## Data Availability

Project name: CDE Semantic Model Implementations. Description: Parent GitHub repository for individual sub-components that generate the YARRRML templates; The YARRRML templates themselves, plus sample CSV data and CSV documentation; and the docker-compose repository used by “CDE in a Box”. Project home page: https://github.com/ejp-rd-vp/CDE-semantic-model-implementations Operating system(s): Platform independent. License: MIT (all subcomponents). Project name: CDE Semantic Model. Description: Diagrams, ShEx, and sample RDF for each of the Semantic Models. Project home page: https://github.com/ejp-rd-vp/CDE-semantic-model Operating system(s): Platform independent. License: MIT. Project name: CDE in a Box. Description: Bootstrap and Production docker-compose files to run CDE in a Box. Project home page: https://github.com/ejp-rd-vp/cde-in-box Operating system(s): Platform independent. License: Apache 2.0. Project name: FAIR in a Box. Description: A fork from CDE in a Box (above) that takes a different approach to installing and orchestrating the docker images. Project home page: https://github.com/markwilkinson/FAIR-in-a-box Operating system(s): Platform independent. License: Apache 2.0.

## Abbreviations

API: Application Programming Interface
BYODs: Bring Your Own Data workshops
CDE: Common Data Element
CSV: Comma Separated Values
EJP RD: European Joint Programme on Rare Disease
ERN: European Reference Network
ETL: Extract, Transform, Load
FAIR: Findable, Accessible, Interoperable, Reusable
GUID: Globally Unique Identifier
OMIM: Online Mendelian Inheritance in Man
OWL: Web Ontology Language
PROM: Patient Reported Outcome Measures
PROV-O: Provenance Ontology
RD: Rare disease
RDF: Resource Description Framework
RML: RDF Mapping Language
ShEx: Shape Expression
SIO: SemanticScience Integrated Ontology
URI: Uniform Resource Identifier
YAML: YAML Ain’t Markup Language
